# ’Make exercise the elixir across an economic divide’: A message to COVID-19 decision makers

**DOI:** 10.17159/2078-516X/2020/v32i1a8310

**Published:** 2020-01-01

**Authors:** J Patricios, R Saggers, B Gelbart, J van Zuydam

**Affiliations:** 1 Wits Institute for Sport and Health (WISH), School of Therapeutic Medicine, Faculty of Health Sciences, University of the Witwatersrand, Johannesburg, South Africa; 2 Department of Paediatrics, School of Clinical Medicine, Faculty of Health Sciences, University of the Witwatersrand, Johannesburg, South Africa; 3 Department of Orthopaedics, School of Clinical Medicine, Faculty of Health Sciences, University of the Witwatersrand, Johannesburg, South Africa

**Keywords:** coronavirus, immunity, exercise, metabolic syndrome

## Abstract

South Africa, like other countries around the world, has used a lockdown strategy to address the initial phases of the COVID-19 epidemic. The significant restrictions on population movement have included initially limiting exercise to the home. There is substantial evidence for the many benefits of exercise. This study specifically emphasises the benefits of exercise to the immune system, particularly for those most vulnerable to the effects of the SARS-CoV-2 virus and proposes measures to improve access to exercise in a demographically diverse and economically disparate society.

South Africa’s lauded response to the global pandemic was initiated publicly by the launch of its hotline to the National Institute of Communicable Disease (NICD) on 14^th^ February 2020, followed by its first recorded case of COVID-19 on the 5^th^ March 2020.^[1]^

## Discussion

### Demographic and health challenges

The country has a demographically heterogeneous population but also a large disparity in resource distribution with a GINI coefficient of 63.^[2]^ While some members of the population live in First World conditions with spacious dwellings, private medical insurance and access to a range of technologies, the majority live in informal settlements, which are densely populated, making the spread of infectious disease more probable and physical distancing challenging. Moreover, South Africa has an HIV prevalence of 20.4% (7.7m people) ^[3]^ and approximately 530 000 tuberculosis cases.^[4]^ Significantly, rates of obesity and metabolic syndrome are also alarmingly high across both cohorts. ^[5]^

### Lifestyle diseases

The published data show that pre-existing conditions, such as obesity, diabetes, hypertension and chronic cardiovascular disease, significantly increase mortality in COVID-19 patients. ^[6,7]^ Just as intensivists know which pre-existing ailments raise a red flag for the COVID-19 patient, Sport and Exercise Medicine clinicians appreciate how regular exercise is a most effective intervention across the spectrum of these comorbidities.

### Pandemic predictions

Initial predictions indicated a potential positive number of South African COVID-19 cases in excess of 12 million by 10 April 2020 ^[8]^, with a 15% incidence of severe disease and a mortality rate of 2%.^[9]^ A significantly lower disease incidence trajectory was achieved by a 5-week hard lockdown that allowed for only essential services to be open and during which exercise outside of homes was prohibited. Not only did economic productivity inevitably decline but so too did levels of physical activity.

### Exercise as the ultimate immune booster

The virtues of regular exercise, namely physical, cognitive, and social, have been validated and extolled repeatedly. Together with sound nutrition, its role in controlling the metabolic syndrome is critical. Less well publicised is the impactful role of exercise on the immune system.

Acute exercise stimulates the interchange of innate immune system cells between lymphoid tissues and the bloodstream. ^[10]^ Over time, a summation effect occurs resulting in improved immunosurveillance against pathogens and decreased systemic inflammation. ^[11]^ Randomised clinical trials have shown how near-daily moderate activity decreases the incidence of upper respiratory tract infections by 40%–50% surpassing the efficacy of most drugs and supplements. ^[12]^

Importantly, a recent systematic review of the immunology literature has shown that the most vulnerable patients with an adverse outcome after SARS-CoV-2 infection may benefit most from the immune-regulating effects of regular exercise.^[11]^ These include obese, diabetic, hypertensive and cardiac patients. Moreover, regular exercisers have a lower incidence and mortality for influenza and pneumonia. There is also an enhanced response to vaccinations, and immunosenesence (the immune dysregulation associated with aging) is delayed by habitual exercise. ^[11]^

Exercise needs to be undertaken gradually by those who are unaccustomed to regular exercise and those who have taken a break from training. Changes in training load should be in small increments, with data indicating that weekly increments should be <10%. ^[13, 14]^

When introduced responsibly, exercise is an immune booster par excellence with a particularly positive effect in those with comorbidities and in age groups commonly associated with poor outcomes to COVID-19. Exercise should be a key intervention rather than a prohibition in any national pandemic strategy. It is also freely available.

### Exercise and risk mitigation

South Africa’s initial lockdown directive was to prohibit not only organised sport, but exercise outside of the home, the goal being to prevent the social interaction associated with sport. Those with the self-discipline and the means (smartphones,

WiFi and space) were able to make use of a number of online resources, including personal trainers and ‘Zoom’ classes. A major health insurer (Discovery) introduced the concept of a home workout channel, “Vitality at Home” ^[15]^, while a Wits Institute for Sport and Health collaboration has produced an online tool (available at https://www.insightfit.com/) to screen athletes for any symptoms of COVID-19 before calculating an individualised safe return to training. But what options exist for those in informal dwellings in an over-populated settlement?

The first relaxation of lockdown restricted exercise to walking, jogging and cycling for a window of three hours in the early morning within a five km radius of home whilst emphasising physical distancing instructions. This proved difficult as socially-starved active communities often turned out en masse counter to the intention, crowding popular public thoroughfares.

### An alternative strategy

If acknowledgement that the immune-boosting benefits of exercise are substantial is given, these authors strongly suggest prioritising the optimisation of immune health during a viral pandemic where the body’s innate and adaptive immune responses are the ultimate defence against the microbe.

A more sophisticated strategy is proposed that sees physical activity prioritised at every stage of risk mitigation against the disease. Specifically:

- Ongoing hygiene measures - handwashing, sanitiser use, respiratory etiquette and increased physical distancing (potentially greater than the two metres recommended for static distancing) during exercise remain non-negotiable. The use of masks and buffs when sharing any public space, including during exercise, seems a sensible (if not scientifically-proven) intervention
- Availing and martialling of public spaces to facilitate exercise with physical distancing under supervised conditions
- Allowing more extended periods of exercise outside of the home to diminish the number of people exercising at once
- Permitting participation in those sports that easily facilitate physical distancing, such as golf, singles tennis, surfing, swimming in open water or in large, chlorinated pools with lanes
- Providing specific guidelines to schools to encourage exercise on school grounds with the return to schools
- Proceeding with a phased introduction of individual and team sports in controlled environments until herd immunity is achieved or a vaccine becomes available.

### Use the opportunity...and this medicine

Physical exercise is accessible across ages, economic strata and social divides. Facilitating outdoor activity in the context of social lockdown may seem counter-intuitive. However, the overwhelming evidence for the benefits of regular physical activity on the human immune system, as well its efficacy as an intervention in those most vulnerable, makes it imperative that exercise is prioritised at every level of lockdown risk stratification. A more nuanced and sophisticated strategy by governments will utilise the current trying global health crisis to highlight exercise as the preventative and interventional medicine that it is.
